# Performance of Aerobic Denitrification by the Strain *Pseudomonas balearica* RAD-17 in the Presence of Antibiotics

**DOI:** 10.3390/microorganisms9081584

**Published:** 2021-07-26

**Authors:** Yunjie Ruan, Lei Cai, Huifeng Lu, Meng Zhang, Xiangyang Xu, Wenbing Li

**Affiliations:** 1 Institute of Agricultural Bio-Environmental Engineering, College of Bio-Systems Engineering and Food Science, Zhejiang University, Hangzhou 310058, China; ruanyj@zju.edu.cn; 2 Academy of Rural Development, Zhejiang University, Hangzhou 310058, China; 3 Laboratory of Microbial Resources, College of Food Science and Biotechnology, Zhejiang Gongshang University, Hangzhou 310035, China; cailei@zjgsu.edu.cn; 4 Zhejiang Water Healer Environmental Technology Co., Ltd., Hangzhou 311121, China; luhuifeng@zju.edu.cn; 5 Department of Environmental Engineering, Zhejiang University, Hangzhou 310058, China; zhang.meng@ntu.edu.sg (M.Z.); xuxy@zju.edu.cn (X.X.); 6 Advanced Environmental Biotechnology Centre, Nanyang Environment & Water Research Institute, Nanyang Technological University, Singapore 639798, Singapore; 7 College of Life and Environmental Sciences, Hangzhou Normal University, Hangzhou 311121, China

**Keywords:** aerobic denitrification, ciprofloxacin, oxytetracycline, strain growth characteristics, N_2_O production

## Abstract

Aerobic denitrification, one of the important nitrate metabolic pathways in biological denitrification, has been attracting increasing interest recently due to its functional advantages. In order to evaluate the effect of antibiotics on aerobic denitrification and guide practical engineering application of aerobic denitrification techniques, we evaluated the performance of aerobic denitrification by the strain *Pseudomonas balearica* RAD-17 in the presence of ciprofloxacin (CFX) and oxytetracycline (OTC). No significant negative impact on the performance of aerobic denitrification in the presence of CFX or OTC within the range of 50 to 300 μg L^−1^ was found. Significant degradation of OTC was found within the range of 50 μg L^−1^ to 300 μg L^−1^ under aerobic denitrification conditions, while no degradation was found for CFX. Stimulation of cell growth occurred within the investigated range of antibiotics. Under anoxic or aerobic conditions, the addition of CFX or OTC changed the N_2_O production trend. The results in the present study may play an important role in informing the use of aerobic denitrification techniques in the presence of antibiotics within environmentally relevant concentrations (<1 mg/L).

## 1. Introduction

Denitrification is a crucial process for nitrogen cycling process in ecosystems, especially for the removal of nitrogen from wastewater [1]. In the past, denitrification mediated by microorganisms is usually considered as a process under anoxic conditions, since the gene encoded for nitrate reductase is sensitive for oxygen [2]. In 1984, Robertson and Kuenen found that denitrification can be achieved under aerobic conditions by aerobic denitrifiers [3]. Due to its functional advantages, aerobic denitrification is an important nitrate metabolic pathway for biological denitrification and has been paid more attention by scientists [4]. Compared to anoxic denitrifying microbe, a gene cluster of *napFDAGHBC* was found in aerobic denitrifying microbe, and this made the nitrate removal process for aerobic denitrifying microbe is different from anoxic denitrifying microbe [5]. As a result, there will be a wider niche and fitness for aerobic denitrification process than anaerobic denitrification process.

Antibiotic overuse, however, is now a common phenomenon and has led to myriad problems [6]. For instance, the spread of antibiotic-resistant bacteria (ARB) and corresponding antibiotic resistance genes (ARGs) has become a worldwide health issue [7]. Tetracyclines (TCs) are one of the most widely used classes of antibiotics, both for human and veterinary treatment, due to their low price, favorable pharmacokinetic characteristics, and the width of the antibacterial spectrum [8,9]. Due to their low utilization rate by humans and animals, 60% to 90% of TCs enter the environment by the excrement of parent compounds or metabolites [10]. As a result, TCs have been detected in various matrices, including soil and sediment [8,11], sewage sludge [12], wastewater [13], surface water [14], and groundwater [15,16]. Therefore, TC resistance (tet) genes are frequently detected in the environment, especially in sewage sludge, during the treatment of municipal wastewater [7]. 

Antibiotics are one of the most complex factors impacting the denitrification process. effects of antibiotics (such as TCs) on denitrification processes have attracted scientific attention. Research on the effect of TCs on denitrification is very limited and has mainly focused on the matrices of bioreactors [17,18,19,20], wastewater treatment plants (WWTPs) [21], and activated sludge [18,20]. However, contradictory conclusions were found for these research [22,23]. The effect of TCs on denitrification is usually dose-dependent, and most studies have demonstrated that denitrification is unaffected by TCs present at environmentally relevant concentrations [1]. While other researcher found that denitrifying genes *nirK* and *nosZ* can be inhibited by TCs at low concentrations (10^−3^ mg L^−1^) [24]. As a result, accumulation of N_2_O was found during denitrification process due to the inhibition of *nosZ* by TCs. In the past, more attention was paid for the effect of antibiotics on anoxic denitrification process. Effect of antibiotics on the performance of aerobic denitrification by pure strain has not gained enough attention.

In the present research, the strain *Pseudomonas balearica* RAD-17, isolated by us from a PBS (poly-butylene succinate) based denitrification reactor used for aquaculture effluent treatment, was employed to investigate the performance of aerobic denitrification of RAD-17 in the presence of ciprofloxacin (CFX) and OTC. The purpose of this research was (1) to evaluate the performance of aerobic denitrification with CFX or OTC; (2) to investigate the growth behavior of RAD-17 and the effect of CFX or OTC on the process of aerobic nitrogen; and (3) to elucidate the mechanism of interaction between nitrate and the strain RAD-17, and then guide practical engineering applications of aerobic denitrification technique.

## 2. Materials and Methods

### 2.1. Basic Information Regarding the Strain RAD-17

The strain RAD-17, employed in the current research, was isolated from a PBS (poly-butylene succinate) based denitrification reactor for the treatment of aquaculture effluent. The operating conditions for the reactor can be found in [22], and information on the isolation and identification of this strain is available in our previous report [23]. The strain RAD-17 was identified as *Pseudomonas balearica* (*P. balearica*), based on 16S rRNA phylogenetic analysis with the Accession Number NCB: MK881511. The strain RAD-17 is a gram-negative bacterium that is 0.3–0.4 μm in diameter and 0.8–1.6 μm in length. In addition, according to the complete genome sequence results (NCBI: PRJNA599547), gene annotation, and KEGG pathway results, the strain RAD-17 embodies a series of genes for denitrification under aerobic conditions. Under aerobic conditions, nitrate-nitrogen (NO_3_^−^–N) and nitrite nitrogen (NO_2_^−^–N) can be removed at rates of 6.22 mg L^−1^ h^−1^ and 6.30 mg L^−1^ h^−1^, respectively [23]. Furthermore, the maximum nitrate reduction rate can be as high as 21.7 mg L^−1^ h^−1^ [25].

### 2.2. Performance of Aerobic Denitrification with Antibiotics

To evaluate the performance of aerobic denitrification for strain RAD-17 with co-existing antibiotics, a batch experiment was carried out under laboratory conditions; this is described here briefly. First, the seed solution was prepared and incubated for 48 h. Then, a seed solution of 1% (*v*/*v*) was inoculated into denitrification media (DM) for nitrogen removal evaluation in 250 mL Erlenmeyer flasks (100 mL solution). The DM was prepared at the ratio of KNO_3_ (sole nitrogen substance, NO_3_^−^-N concentration 300 mg/L), sodium acetate (sole organic-carbon substance, C/N ratio fixed on 10), 0.2 g/L MgSO_4_·7H_2_O, 1.0 g/L K_2_HPO_4_, and 1.0% (*v*/*v*) trace-element solution. The trace-element solution contained 50.0 g/L ethylenediaminetetraacetic acid (EDTA), 2.2 g/L ZnSO_4_, 5.5 g/L CaCl_2_, 5.06 g/LMnCl_2_·4H_2_O, 5.0 g/L FeSO_4_·7H_2_O, 1.1 g/L (NH_4_)_6_Mo_7_O_2_·4H_2_O, 1.57 g/L CuSO_4_·5H_2_O, 25 g/L NaCl and 1.61 g/LCoCl_2_·6H_2_O [8]. Two typical antibiotics, CFX and OTC, were employed in the form of hydrochloride, and a concentration series was set up at 0, 50, 100, 150, 200, and 300 μg L^−1^. To exclude abiotic degradation, no inoculation was carried out for the DM containing 300 μg L^−1^ CFX or OTC and served as the abiotic degradation control. Before and after 48 h, under incubation conditions of 150 rpm at 30 °C, the concentration of total organic carbon (TOC), NO_3_^−^-N, NO_2_^−^-N, NH_4_^+^-N, and CFX or OTC were determined. There were three replicates for this experiment. Moreover, dynamic degradation experiments of CFX or OTC were also carried out with the highest antibiotic concentrations (300 μg L^−1^). During this experiment, the concentration of CFX or OTC at 12, 24, and 48 h was determined, and five replicates for each treatment were employed. Detailed information on the CFX or OTC analysis procedures can be found in our recently published reports [8].

### 2.3. Flow Cytometry Analysis Experiment

To evaluate the variation of strain cell numbers, a flow cytometry analysis experiment was carried out. After pre-culturing in DM, the strain RAD-17 was inoculated into new DMs (1%, *v*/*v*), which contained 0 μg L^−1^ and 300 μg L^−1^ of CFX or OTC before being incubated under aerobic conditions. The strain solutions were sampled at 12 h, 24 h, and 48 h and were diluted with sterile saline solution (NaCl, 25‰) to reach a cell density of 1.0 × 10^6^ cells ml^−1^ to 1.0 × 10^7^ cells ml^−1^. Afterward, 0.5 mL of diluted strain solution was sampled, and then the determination was carried out by flow cytometry (FACSMelody, BD, Franklin Lakes, NJ, USA). Detailed information on the calculation of the strain cell density can be found in our recent publications [25]. In order to guarantee the accuracy of the experimental results, 15 replicates for each treatment were used.

### 2.4. Detection of N_2_O Gas

After pre-culturing in DM for 48 h at 30 °C, 0.5 mL of strain solution was inoculated into 50 mL medium in 100 mL headspace vials (with rubber stoppers), which contained 0 μg L^−1^ and 300 μg L^−1^ of CFX or OTC, respectively (*n* = 10). After incubation for 48 h at 30 °C under aerobic or anaerobic conditions, 5 mL of the headspace product was sampled using a 25 mL syringe for N_2_O analysis by gas chromatography (GC-2010 Plus SHIMADZU, Kyoto, Japan). Detailed information on the analysis procedure can be found in our recently published reports [8].

### 2.5. Chemical Analysis

Samples for chemical analysis were first filtered through a 0.45 μm filter membrane, and then the determination of NO_3_^−^-N, NO_2_^−^-N, and NH_4_^+^-N followed the standard methods [26]. TOC and cell growth (OD_600_) were determined by a TOC analyzer (Multi N/C 2100, Analytik Jena, Jena, Germany) and spectrophotometer at 600 nm, respectively. In addition, the inhibition rate of the net OD_600_ value was also calculated by the following formula:

Inhibition rate of the net OD_600_ value = (Cell_CK_ − Cell_Treatment_)/Cell_CK_ × 100%

### 2.6. Statistical Analysis

The statistical analysis was performed using SPSS 20.0 (SPSS Inc., Chicago, 179 IL, USA). In order to evaluate the difference between different treatments, a one-way analysis of variance was employed.

## 3. Results

### 3.1. Aerobic Denitrification Performance in the Presence of Antibiotics

**Figure 1:**
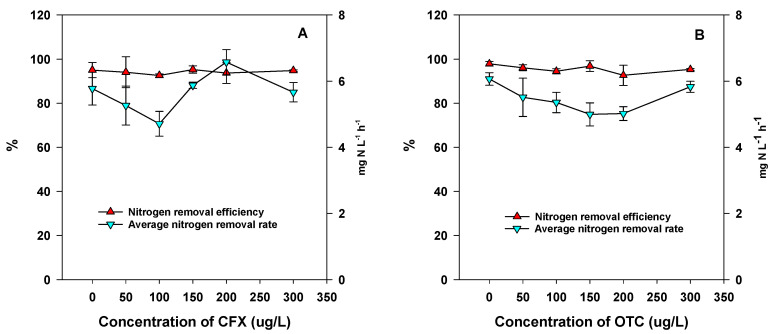
Aerobic denitrification performance in the presence of CFX (**A**) and OTC (**B**).

In the presence of CFX, there were no significant impacts on the aerobic denitrification performance of strain RAD-17 within the range of 50 μg L^−1^ to 300 μg L^−1^ (Figure 1A). The nitrate removal efficiency varied from 92.57 ± 0.75% to 95.27 ± 1.67%, which was similar to the results obtained without CFX addition (95.00 ± 3.40%). Furthermore, the average nitrogen removal rate (6.58 ± 0.38 mg NO_3_^−^-N L^−1^ h^−1^) in the presence of 200 μg L^−1^ was significantly higher than all the other treatments. In the presence of OTC, there were also no significant impacts on the aerobic denitrification performance of strain RAD-17 within the range of 50 μg L^−1^ to 300 μg L^−1^ (Figure 1B). The nitrate removal efficiency varied from 92.65 ± 4.56% to 96.82 ± 2.40%, which was slightly lower than the results obtained without OTC addition (97.88 ± 1.07%). 

### 3.2. Degradation Behavior of Antibiotics under Aerobic Denitrification Conditions

**Figure 2:**
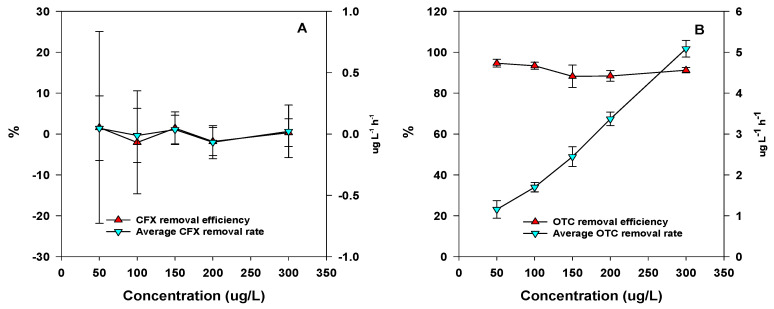
Degradation of CFX (**A**) and OTC (**B**) in the aerobic denitrification process.

Under aerobic denitrification conditions, there was no degradation of CFX within the range of 50 μg L^−1^ to 300 μg L^−1^, and the results of parallel samples varied greatly (Figure 2A). However, significant degradation of OTC was found within the range of 50 μg L^−1^ to 300 μg L^−1^ under aerobic denitrification conditions (Figure 2B). The degradation efficiency of OTC was 88.23 ± 5.52% to 94.63 ± 1.88%, with initial concentrations ranging from 50 to 300 μg L^−1^. Furthermore, the average OTC removal rate increased with the increase of the OTC concentration. The average OTC removal rate was 1.15 ± 0.22 μg OTC L^−1^ h^−1^ when the initial OTC concentration was 50 μg L^−1^. However, the average OTC removal rate was as high as 5.09 ± 0.21 μg OTC L^−1^ h^−1^ when the initial OTC concentration was 300 μg L^−1^. 

### 3.3. Degradation Behavior of TOC under Aerobic Denitrification Conditions

**Figure 3:**
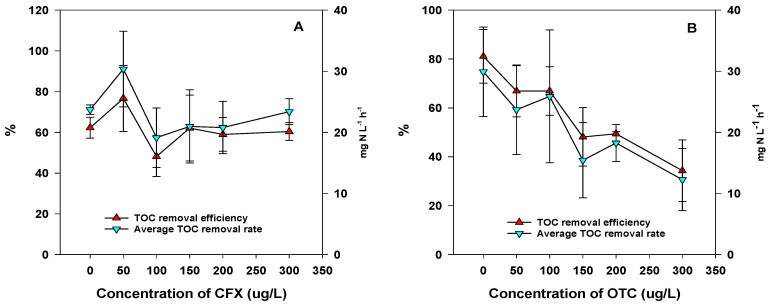
Variation of TOC under aerobic denitrification processes in the presence of CFX (**A**) and OTC (**B**).

Under aerobic denitrification conditions, variation of TOC degradation was found between 48.15 ± 9.86% to 76.69 ± 16.23% in the presence of CFX, within the range of 50 μg L^−1^ to 300 μg L^−1^ (Figure 3A). In addition, the average TOC removal rate varied from 19.13 ± 4.86 mg L^−1^ h^−1^ to 30.36 ± 6.20 mg L^−1^ h^−1^. In the presence of OTC, TOC degradation decreased with the increase of the OTC concentration from 0 μg L^−1^ to 300 μg L^−1^ under aerobic denitrification conditions (Figure 3B). Without OTC addition, TOC degradation was as high as 81.08 ± 10.99%. However, TOC degradation decreased from 66.87 ± 10.48% to 34.30 ± 12.65% when the OTC concentration increased from 50 μg L^−1^ to 300 μg L^−1^. Moreover, the average TOC removal rate decreased from 29.92 ± 7.33 mg L^−1^ h^−1^ to 12.26 ± 5.08 mg L^−1^ h^−1^.

### 3.4. Growth Behavior of Strain RAD-17 in the Presence of Antibiotics

**Figure 4:**
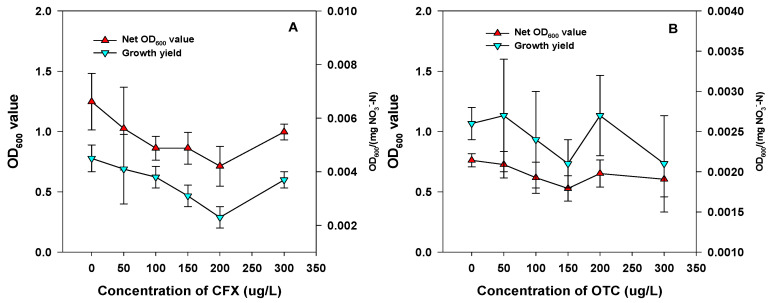
Growth behavior of strain RAD-17 in the presence of CFX (**A**) and OTC (**B**).

In order to evaluate the impact of antibiotics on the behavior of strain RAD-17, the changes of the OD_600_ value and cell numbers were employed in the concentration change experiment and dynamic change experiment. In the presence of CFX, there was a slight inhibition of the growth of strain RAD-17 (Figure 4A). Without CFX addition, the net OD_600_ value and growth yields were 1.25 ± 0.23 and 4.50 ± 0.50 × 10^−3^ OD_600_/(mg NO_3_^−^-N), respectively. However, the net OD_600_ value varied from 0.71 ± 0.16 to 1.02 ± 0.34 with the addition of 50 to 300 μg L^−1^ of CFX. Accordingly, the net OD_600_ value and growth yield varied from 2.30 ± 0.40 × 10^−3^ OD_600_/(mg NO_3_^−^-N) to 4.10 ± 0.13 × 10^−3^ OD_600_/(mg NO_3_^−^-N). In the presence of OTC, a more serious impact on the growth of strain RAD-17 was found than in the presence of CFX (Figure 4B). The net OD_600_ value and growth yield were 0.76 ± 0.06 and 2.60 ± 0.20 × 10^−3^ OD_600_/(mg NO_3_^−^-N), respectively. In addition, the net OD_600_ value varied from 0.53 ± 0.10 to 0.73 ± 0.11 with the addition of 50 to 300 μg L^−1^ of OTC. Accordingly, the net OD_600_ value and growth yield varied from 2.10 ± 0.30 × 10^−3^ OD_600_/(mg NO_3_^−^-N) to 2.70 ± 0.70 × 10^−3^ OD_600_/(mg NO_3_^−^-N).

**Figure 5:**
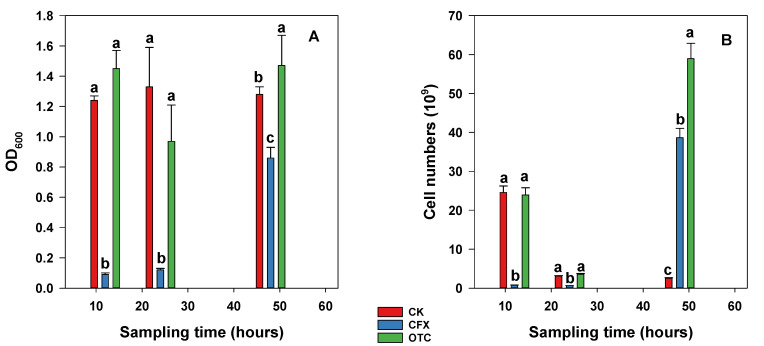
Variation of cell numbers (**A**) and OD600 value (**B**) with different treatments. Columns in (**A**,**B**) containing different letters indicate significant differences among concentrations of the same antibiotics at *p* = 0.05.

Furthermore, the dynamic changes of the net OD_600_ value and cell numbers were also investigated with the addition of CFX and OTC. Without antibiotic treatment, no significant change was found in a 12 h to 48 h cultivation period for the net OD_600_ value, which varied from 1.24 ± 0.03 to 1.33 ± 0.26 (Figure 5A). However, the cell numbers decreased from 24.6 ± 0.77 × 10^9^ to 2.55 ± 0.11 × 10^9^ in the 12 h to 48 h cultivation period (Figure 5B). With the addition of antibiotics, different dynamic changes occurred in net OD_600_ value and cell numbers. In the presence of CFX, there was no significant difference for the net OD_600_ value and cell numbers in the 12 h to 24 h cultivation period. However, the net OD_600_ value and cell numbers increased up to 0.86 ± 0.07 and 38.65 ± 2.39 × 10^9^ after 48 h cultivation. Similar dynamic change behavior for the net OD_600_ value and cell number was also found for the treatment with OTC.

### 3.5. Effect of Antibiotics on the Denitrification Process

**Figure 6:**
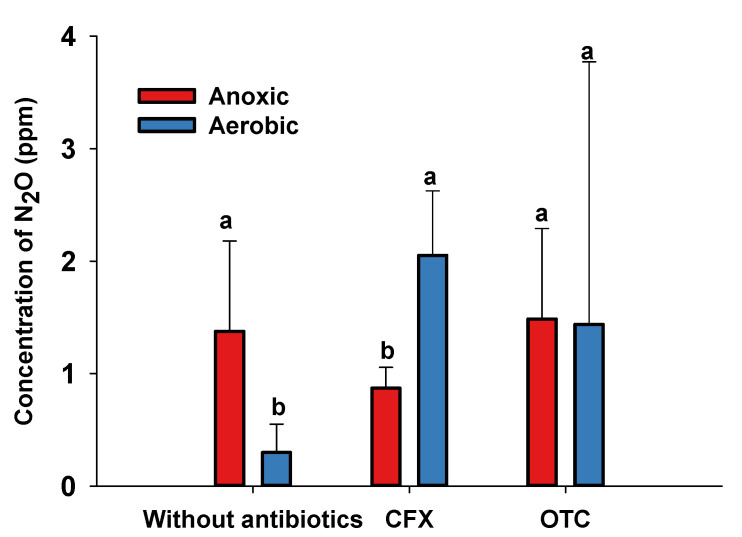
Concentration of nitrous oxide (N_2_O) under aerobic and anoxic conditions for strain RAD-17 with different treatments. Columns containing different letters indicate significant differences among concentrations of the same antibiotic at *p* = 0.05.

Nitrous oxide (N_2_O) was determined to evaluate the effect on the denitrification process of CFX and OTC, and different trends were found among the three treatments (Figure 6). Without the addition of antibiotics, the N_2_O content under anoxic conditions (1.38 ± 0.80 ppm) was significantly higher than that under aerobic conditions (0.30 ± 0.25 ppm) (*p* = 0.05). The opposite trend was observed in the presence of CFX; the N_2_O content under aerobic conditions (2.05 ± 0.57 ppm) was significantly higher than that under anoxic conditions (0.87 ± 0.19 ppm) (*p* = 0.05). However, no significant change was found for the N_2_O content between aerobic conditions (1.44 ± 2.22 ppm) and anoxic conditions (1.49 ± 0.80 ppm).

## 4. Discussion

### 4.1. Aerobic Denitrification Performance 

In the current research, no significant impacts on the aerobic denitrification performance of strain RAD-17 were found within the range of 50 μg L^−1^ to 300 μg L^−1^ for both CFX and OTC. the nitrate removal efficiency was similar to previous reports on strain RAD-2, which also maintained a high level of removal efficiency in the presence of antibiotics [8]. Furthermore, the average nitrogen removal rate decreased with the increase of OTC concentration, and all the treatments (with the addition of OTC) were significantly lower than the control (6.07 ± 0.19 mg NO_3_^−^-N L^−1^ h^−1^) (*p* = 0.05). The nitrate removal rates of RAD-17 were in accordance with previous reports on pure strains, with or without addition of antibiotics, e.g., 6.47 mg NO_3_^−^-N L^−1^ h^−1^ (without antibiotics) and 5.72 to 6.46 mg NO_3_^−^-N L^−1^ h^−1^ (in the presence of antibiotics) for strain RAD-2 [2], and 6.22 mg NO_3_^−^-N L^−1^ h^−1^ for strain RAD-17 (without antibiotics) [23]. As mentioned above, effect of antibiotics on denitrification is dose dependent. For anoxic denitrification process experiment by Feng et al. (2020), who found that nitrate removal efficiency maintained high level under low level OTC stress (<1 mg L^−1^) and decreased significantly under high level OTC stress (5 mg L^−1^) [27]. Similar results were also found by Zhu et al. (2017), who reported that significant nitrate accumulation occurred in the presence of 2 mg L^−1^ TC [28].

### 4.2. Degradation Behavior of Antibiotics

In the current research, there were high removal efficiency for OTC under aerobic denitrification process. Physical and chemical processes are commonly used abiotic techniques for the removal of TCs, and include filtration, adsorption, and oxidation [2,6,7]. Among the biotic techniques, microbial degradation of TCs is a preferred process, owing to its efficiency and low cost [29]. Degradation of TCs by fungus is another commonly used method and has been reported in some literature. For instance, *Pleurotus ostreatus* mycelium was found to remove Oxytetracycline (OTC) completely within 14 days [16]. In addition, some other pure fungi have been isolated from various matrices and have also been proven to degrade different types of TCs under aerobic conditions, including Trichosporon mycotoxinivorans XPY-10 [30], *Trichoderma deliquescens* RA114, *Penicilium crustosum* RA118 [31], *Fusarium* sp. CMB-MF017, and *Paecilomyces* sp. CMBMF010 [32]. In comparison, only a few pure bacteria strains have been isolated for the degradation of TCs. After a 27 h incubation period, nearly 50% of the TCs (20 mg L^−1^) were degraded by thestrain *Sphingobacterium* sp. PM2-P1-29, which contains the tet (X) gene [33]. In addition, the *Stenotrophomonas* strain was isolated from soil, and has been shown to capable of degrading 44.43 mg L^−1^ of TCs within four days [15].

### 4.3. Research Implicaitons

In the current research, high removal efficiency for aerobic denitrification were achieved in the presence of CFX or OTC. Furthermore, simultaneous aerobic denitrification and OTC degradation was found. However, before application of aerobic denitrification techniques in the presence of antibiotics, satisfied solution needs to be searched for the following issues: (1) aerobic denitrification pathways should be clearly understood. In the presence of antibiotics, expression of denitrifying genes (e.g., *napA*, *nirs*, *norB*, and *nosZ*) and the direct receptor genes should be investigated, and this is useful to understand aerobic denitrification behavior. For instance, different concentration of N_2_O were found under aerobic and anoxic conditions, and this might be related to expression of *nosZ*. (2) Cocktail effects of antibiotics to aerobic denitrification. In the current and some other related studies, single type of antibiotics was employed for the effect of antibiotics on aerobic denitrification. In a more realistic situation, multiple types of antibiotics rather than single type antibiocis were found. Thus, synergy or antagonism effect among multiple types of antibiotics should be also investigated in future. 

## 5. Conclusions

There was no significant influence on the aerobic nitrate removal efficiency of strain RAD-17 with simultaneous denitrification and antibiotic degradation, regardless of the antibiotic concentration. Significant degradation of OTC was found within the range of 50 μg L^−1^ to 300 μg L^−1^ under aerobic denitrification conditions, while no degradation was found for CFX. Stimulation of cell growth might occur within the investigated range of antibiotics. Under anoxic or aerobic conditions, the addition of CFX or OTC changed the N_2_O production trend. In the future, it is necessary to investigate the effect of antibiotics on the metabolic pathway by metagenome and macrotranscriptome methods and find the crucial genes involved in the denitrification-related pathway.
